# Ten simple rules for establishing an experimental lab

**DOI:** 10.1371/journal.pcbi.1011778

**Published:** 2024-01-25

**Authors:** Marcus Kaiser

**Affiliations:** 1 Precision Imaging, School of Medicine, University of Nottingham, Nottingham, United Kingdom; 2 NIHR Nottingham Biomedical Research Centre, School of Medicine, University of Nottingham, Nottingham, United Kingdom; 3 Department of Neurosurgery, Ruijin Hospital, Shanghai Jiao Tong University School of Medicine, Shanghai, China; Dassault Systemes BIOVIA, UNITED STATES

Computational researchers often collaborate with experimental researchers, but what about starting their own experimental lab alongside computational work? While collaborating with experimental colleagues might be beneficial, maybe you have ideas for experiments that have not been done or maybe you want to measure parameters that would be useful for models, but that have not been obtained in experimental studies? Or maybe you want to simply test whether the predictions from your computational models are true for biological systems?

The move to experimental studies might occur at different career stages. After completing a theoretical or computational undergraduate degree or PhD, one might move into an experimental lab. However, moving into experimental work becomes more challenging when moving from a career and track record that is solely based on computational research. Here, we share *Ten Simple Rules* in an attempt to make this transition feasible at later career stages.

Moving to experimental studies can be daunting for several reasons. Managing an experimental lab is different from managing a computational lab. While data exists in cyberspace, devices in the real-world need lab space, maintenance, and technical support. Also, experiments involve ethics proposals and dealing with animals or with human participants. Finally, moving to experimental research can elicit discouraging thoughts such as “How will I get funding without a track record in experimental research,” “How will I manage to start my career from scratch again,” and “How will I find time to learn what is needed?”. Doing experimental research has a steep learning curve and is a major commitment; it is not right for everyone.

However, combining computational and experimental research in your lab can be extremely rewarding. Hodgkin and Huxley not only performed mathematical modelling of neural activity (action potentials), but also developed experiments to get quantitative data about the phenomenon [1]. It is this unique combination of modelling and experiments that led to insights about action potential and ultimately led to them being awarded the Nobel Prize in Physiology or Medicine (for 10 other ways to win the Nobel Prize, see [2]).

So, you have made the decision to move into experimental research. Now what?

## Rule 1: Link up with experimentalists

If you are thinking about setting up an experimental lab, it is crucial to establish links with other experimental groups to learn about techniques and lab organisation. You might already have research collaborations with experimental labs that can give you information about what equipment to buy and what processes are in place for planning experimental studies.

After closely collaborating with experimental researchers, it is crucial to spend actual time in their lab—not just observing but participating in experiments. Spending more time will give you a better understanding of what can go wrong during experiments and what the particular pitfalls and challenges are. Following your experience in an experimental lab for several weeks or months, potentially enabled through a sabbatical, you will know what you can expect from setting up your own experiments.

For some experimental techniques, especially the ones that will not be used often in your lab, it is more efficient to work with other labs than to establish that technique in your own lab. If you can get data from other labs, this of often easier: also experimental lab collaborate with other experimental labs, they do not do everything themselves. Doing your own experiments is only necessary when you need experiments run that others cannot or will not do for you.

One possibility to get closer links with experimental groups is to get a joint affiliation with experimental departments within your institution. This will also help you to learn about collaboration and funding opportunities within the experimental sciences. Indeed, linking up with other units gives you a chance to attend talks and seminars about experimental topics.

Beyond learning about the design of experiments, it is important to gain an understanding of the underlying concepts in biology, chemistry, or physics that are related to the phenomena that you study. While your graduate or undergraduate degrees might already have contained some relevant modules, you might think about attending modules within master’s or undergraduate degrees, training workshops, or online degree courses to learn in more depth.

## Rule 2: Keep up to date with experimental techniques

It is a good idea to join conferences where experimental work is presented and to join academic societies for experimental research. Unfortunately, many societies are either focused on experimental or computational research. Try to find societies that combine both areas of research. For the British Neuroscience Association, for example, there is a special interest group in Neuroinformatics. Similarly, experimental and computational work is supported within the Society for Neuroscience.

You will need to train students and postdoctoral fellows in your lab so knowing these techniques is crucial. While attending workshops and visiting labs is an option to learn techniques for yourself, an alternative is to hire a postdoc with the relevant experimental skill (maybe someone who is interested in learning some computational skills when coming to your lab). This postdoc can then train you and other members of your lab.

## Rule 3: Find a space for your lab

You might be based in a department or school that does not have experimental labs. Or, experimental labs might not include research on animals or humans. While experimental labs in microbiology, *C*. *elegans* research, or human–computer interaction can be based within Schools of Computing, Mathematics, or Physics, establishing animal or human experimental labs at your place might not be an option. This means that you might need to move to a different department within your university or at another university. Within your institution, you might be able to secure lab space and equipment in a different school or department. This might not be ideal but could be a good starting point.

If you decide to move to a different place, this often involves the two-body problem of also finding an academic position for your partner. In addition to advertised positions, also keep in mind that fellowship holders can often take their fellowship position with them to a new place so trying to get a fellowship for one of you could be an option. In exceptional cases, new positions could also be created for one of you. However, in any case, working at separate places or working remotely will be needed during the transition period.

## Rule 4: Keep computational research ongoing

When you start an experimental lab, should you give up on your computational work? In my opinion, it is a good idea to keep both branches of your research active. Concerning computational skills as your core expertise, you will “use it or lose it” unless you also keep that kind of research going. Computational models can inform experimental studies and experimental data can drive modelling and data analysis. You might want to keep both strands alive through collaborations, attending meetings concerning experimental and computational research, and through visiting positions. As a visiting faculty member in a different institution, you can complement your own research and learn about novel experimental or computational approaches. To keep contacts with theoretical and computational researchers alive, you might be able to share data from your lab for advanced analysis or modelling.

## Rule 5: Get funding for your lab

Experimental labs are expensive with costs for equipment, maintenance, and support not to mention funds for staff, technicians, and PhD students. Depending on your country, funding for experimental and computational research might come from different funders. Also, in general, success rates for biomedical funding are lower and the need for pilot data is higher compared with funding for science, engineering, and computing research. However, there is also a wider range of funders ranging from government and industry to charities related to specific diseases (e.g., cancer research or dementia).

One crucial aspect of funding for biomedical research is often a greater need to present pilot results at the application stage. Such initial findings can suggest the feasibility or some support for the hypothesis of the proposal. If experiments are to feed into computational models, preliminary support for the to be tested hypothesis will be needed. Therefore, look out for smaller funding for pilot experiments and internal funding within your institution. As you will need to show expertise in all areas of a proposal, you might want to include collaborators with a track record of experimentation.

When you approach a biomedical funder, forget everything you think you know about writing successful research grant proposals based on your experience in getting funding in the fields of engineering, mathematics, computing, and physical sciences. Instead, try to find out what style of proposal writing is successful. Looking at the online guidelines for applicants is not sufficient to find out what a funder wants to fund. You can often find a list of funded projects or look at previously successful proposals from colleagues to give you a better idea. You might also get to know the tricks by being coinvestigator on a proposal for that funder. Also, some funders allow you to be an observer for funding panel meetings. Finally, you might have a chance to become a panel member to learn more about the process.

## Rule 6: Keep your lab well organised

You might have organised your code and data on GitHub and the cloud, but experimental equipment exists in physical space. Be sure to set up an electronic inventory (https://www.labmanager.com/lab-inventory-management-guide-12258) of all items early on. Note the purchase date, serial number, and the version number of any firmware: such information will be useful later and help with the reproducibility of your results. In addition, note the location of each item and/or who is using the equipment now. Make sure to make the list available to your team so that this information can be easily updated later. Also, take note of all equipment and parameters that are using during each experiment. Results may differ later when firmware is updated, or new equipment is used.

Familiarise yourself with all the rules and procedures, from storing and handling chemical to dealing with animal tissue or operating equipment (e.g., lasers or MRI scanners).

Maintaining a lab is time consuming, so having a suitable technician (possibly working part-time in your lab at the start) can be extremely helpful.

## Rule 7: Get to know ethics applications

Writing ethics proposals for an experimental study can be a scary thought for a computational researcher. In general, the difficulty depends on the experimental procedure (invasive or noninvasive), the species (animals or humans), and the conditions (healthy subjects or patients). Moreover, there might be other legal requirements in other experimental fields (e.g., the regulation of genetically modified or potentially invasive organisms), depending on local legislations.

Ask colleagues to get samples of previously successful ethics applications and attend training events where possible. For your first study, you might also work with experimental colleagues who can help you with the process.

A critical issue is the number of participants or animals in your study. While you want to recruit enough participants to attain full statistical power, you also want to keep numbers as low as possible for ethical reasons. Whereas there is usually no discussion of whether you need to run a computer model 50 or 100 times, recruitment numbers are crucial for experimental work. Find out about the statistics of planning your experiments and get advice from services within your university (e.g., the clinical trials unit if you run studies in patients). You can use software tools such as G*Power [3] (http://www.psycho.uni-duesseldorf.de/abteilungen/aap/gpower3/) to calculate the statistical power of your experiments. Moreover, there are online tools for planning the overall design of your study such as NC3Rs’ EDA [4] (Experimental Design Assistant: RRID:SCR_017019, https://eda.nc3rs.org.uk). These and other tools will help you to explain clearly why you need a certain number of participants or animals.

## Rule 8: Expect 3 years before your lab is up and running and producing results

Setting up an experimental lab takes time: from securing lab space and equipment to recruiting and training new lab members will take time even if you have managed to obtain funding. Make sure to find out early on about the process for purchasing, ethics applications, and recruitment of study participants.

Be prepared for 3 years before your lab is up and running and first results are published. In the meantime, you might get your previous computational work published. Or you could be involved in manuscripts through research collaborations.

## Rule 9: Lab notebooks and documenting experiments

Keeping the data of experiments is important. But it is also crucial to keep the metadata for each experiment: What devices and parameters were used? What is the version number of hardware, firmware, and software? What are the characteristics of participants? In addition to data, you might want to take photos and videos of setups and experimental procedures to document the work.

You might want to write guidance documents for yourself and your lab on how to store and document data and how experiments should be run (standard operating procedure). Consider also reusing computational tools that you are likely already using for managing reproducible computational workflows (e.g., GitHub versioning, tagging, actions) to manage metadata about your experimental protocols in similar ways. In general, leveraging your experience to develop a lab culture where all metadata is recorded at the point of capture in a fully versioned computational system will pay dividends later, when lab experiments get delayed or lab members move on.

If you involve human participants, there will be special procedures to ensure security of data (e.g., GDPR). Moreover, there might be data handling guidelines from funders and from your institution (e.g., descriptions of what is stored, where, with links to relevant draft and published papers resulting from this data).

If there is a chance that intellectual property arises from the work, you might think about electronic lab notebooks [5,6]. Ensure that access rights are properly managed and make sure that all lab members are trained to keep lab notebooks and to have a system for backups in place.

## Rule 10: Publish in experimental journals

Publishing experimental work can be different from publishing computational studies. If you have a computational component in your work, you might consider journals such as *PLOS Computational Biology*. You can also collaborate with experimental researchers and provide a computational component. Being involved in the publication process will give you a good idea how experimental papers are organised. It is important to read early on about experimental studies to learn about the style of publications in your experimental area. For your first experimental papers, you can follow the style and methodology of recent publications. You might also want to collaborate with experimental colleagues or at least ask them to read, check, and comment on the first manuscripts from your lab.

Overall, the move to set up an experimental lab, especially as a mid-career scientist, is a great adventure. While it is a steep learning curve, you have a chance to directly test predictions from your computational models or to get the ideal data to feed into your analysis or simulation workflows. These Ten Simple Rules are lessons learned over the past 3 years when setting up an experimental lab and witnessed in the transitions of other researchers. As an experimental lab is launched, there is tremendous satisfaction in validating theoretical hypothesis and moving closer to the translation of theory-informed experimental procedures.
